# A Different Method of Hepaticojejunostomy for Proximal Biliary Injuries

**DOI:** 10.1155/1996/79783

**Published:** 1996

**Authors:** Diane M. Radford, Glennon Schaefer

**Affiliations:** 1 Washington University Medical Center 9901 Wohl Hospital 660 South Euclid Ave St. Louis MO 63110 USA; 2 St. John's Mercy Medical Center 615 Ballas Road Suite #196-C St. Louis MO 63141-8277 USA

## Abstract

The management of proximal biliary injuries presents a surgical challenge. Anastomoses can be difficult to perform and can have poor results. We describe a method of hepaticojejunostomy done from within the Roux-en-Y loop, which can be utilized in this situation.


*HPB Surgery*, 1996, Vol.10, pp. 111–112
Reprints available directly from the publisher
Photocopying permitted by license only

© 1996 OPA (Overseas Publishers Association)
Amsterdam B.V. Published in The Netherlands
by Harwood Academic Publishers
Printed in Malaysia

### CASE REPORT
### A Different Method of Hepaticojejunostomy for Proximal Biliary Injuries

DIANE M. RADFORD[1] and GLENNON SCHAEFER[2]

[1]Washington University Medical Center, 9901 Wohl Hospital, 660 South Euclid Ave, St. Louis, MO 63110
St. John's Mercy Medical Center, 615 Ballas Road, Suite #196-C, St. Louis, MO 63141-8277

*(Received 28 October 1993)*

The management of proximal biliary injuries presents a surgical challenge. Anastomoses can be difficult to perform and can have poor results. We describe a method of hepaticojejunostomy done from within the Roux-en-Y loop, which can be utilized in this situation.

KEY WORDS: Biliary Injury bile ducts

### CASE REPORT

A 23-year-old white female underwent cholecystectomy at an out-of-state hospital. Several days postoperatively, she was noted to have bile draining from a Penrose drain, and was referred to St. John's Mercy Medical Center for further management. Percutaneous transhepatic cholangiogram revealed complete transection of the right and left hepatic ducts near the hilum. This finding was confirmed at operation. The injury had occurred just below the exit of the right and left hepatic ducts from the liver, only a few mm of each duct being visible. A large segment of biliary tree was absent, the other end comprising the distal common bile duct, thus making a primary anastomosis impossible.

The repair was performed in the following manner: A Roux-en-Y limb was created and brought up to the hilum. An enterotomy was created, to which the hepatic ducts would be sutured (Figure 1). Another enterotomy was made opposite to the first incision, taking care to avoid the mesentery. The hepaticojejunostomy was created using interrupted, absorbable sutures in a mucosa to mucosa fashion. The anastomosis was sewn from within the bowel and could be visualized easily through the opposite incision (Figure 2). The completed anastomosis is seen in Figure 3. Stents were left in placed in both ducts prior to closure of the other enterotomy.

### DISCUSSION

Bismuth has divided injuries and strictures of the bile ducts into five categories.[1] Destruction of the hilar confluence with separation of the right and the left hepatic ducts is termed a Type 4 injury. The higher the stricture, the more difficult reconstruction becomes and good results become less likely. It is generally agreed that Roux-en-Y hepaticojejunostomy is the best method of reconstruction for high injuries[1-3] Exposure of adequate length of the hepatic ducts can be challenging. Where the injury is so high, as in this case, a liver split may be necessary[1]. The technique described results in good mucosa to mucosa approximation.

Without the need to perform a liver split. Smith has published a similar technique of anastomosis to the common bile duct through the still-open end of the Roux-en-Y[4], but no such technique has been described for injuries to the hepatic duct.

*Correspondence to*: Dr. D.M. Radford Department of Surgery Washington University Medical Center, Suite 6104 Queeny Tower St Louis MO 63110 USA

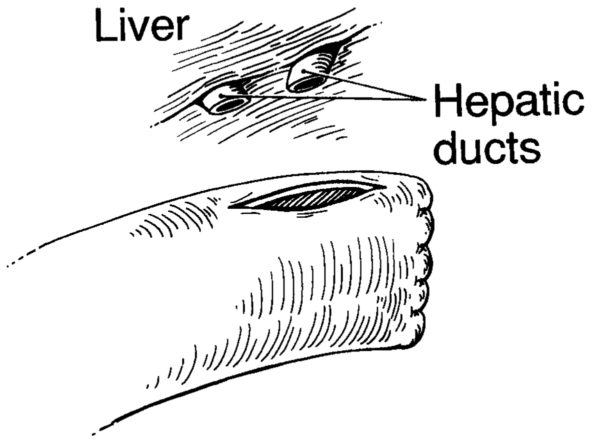

**Figure 1** Enterotomy created in Roux-en-Y line for suture to hepatic ducts.

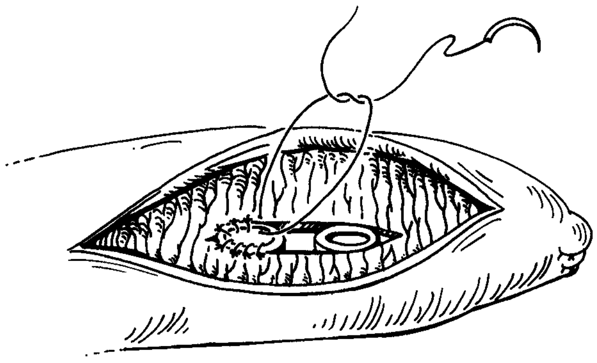

**Figure 2** Second enterotomy created opposite the first enterotomy to allow anastomosis from within the bowel.

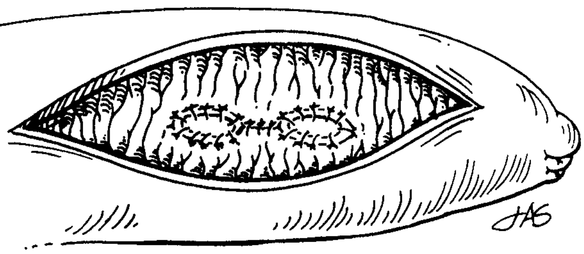

**Figure 3** Completed anastomosis to be followed by closure of the second enterotomy.

### BIBLIOGRAPHY

1. Blumgart L.H, Thompson J.N. (1987) The management of benign strictures of the bile duct. *Curr Prob Surg*, 6–66.
2. Mathisen O. Bergan A. Flatmark A. (1987) Iatrogenic bile duct injuries. *World J Surg* , **11**: 392–97.
3. Browder I.W. Dowling J. Koontz K. Lictwin M.S . (1987)Early Management of operative injuries of the extrahepatic biliary tree. *Ann Surg* , **205**: 649–58.
4. Smith R. (1964) Hepaticojejunostomy: Choledochojejunostomy. A. method of intrajejunal anastomosis. *British J surg* , **51**: 183–86.

